# Exceptional response to everolimus in an NF1-deficient recurrent endometrioid adenocarcinoma: a case report

**DOI:** 10.3389/fonc.2026.1828591

**Published:** 2026-05-25

**Authors:** Maja Machulak, Magdalena Zwierzchowska, Kinga Łysak, Kamil Kośmider, Katarzyna Szklener, Magdalena Skórzewska

**Affiliations:** 1 Student Scientific Society, Department of Clinical Oncology and Chemotherapy, Medical University of Lublin, Lublin, Poland; 2 Department of Internal Medicine and Diabetology, Copernicus Hospital, Gdańsk, Poland; 3 Department of Clinical Oncology and Chemotherapy, Medical University of Lublin, Lublin, Poland; 4 Department of Pneumonology, Oncology and Allergology, Medical University of Lublin, Lublin, Poland

**Keywords:** endometrial cancer, endometrioid adenocarcinoma, everolimus, mTOR, NF1 mutation

## Abstract

Endometrial cancer is among the most commonly diagnosed malignant neoplasms of the female reproductive system, with peak incidence occurring in the perimenopausal and postmenopausal periods. The pathogenesis of this disease is multifactorial and associated with several well-established risk factors, including hormonal imbalance, obesity, and late onset of menopause. We report the case of a 64-year-old woman diagnosed with grade I endometrioid adenocarcinoma, stage IA according to the International Federation of Gynecology and Obstetrics (FIGO) 2009 classification. The patient initially underwent total laparoscopic hysterectomy with bilateral salpingo-oophorectomy. More than three years after surgery, disease recurrence was detected during routine follow-up. Subsequent molecular analysis revealed a loss-of-function mutation in the *NF1* (neurofibromin 1) gene. Based on the molecular profile of the tumor, the patient was qualified for off-label targeted therapy with everolimus, an inhibitor of the mammalian target of rapamycin (mTOR) pathway. Treatment with everolimus resulted in complete remission, which has been sustained to date. However, therapy was associated with significant metabolic toxicity, including severe dyslipidemia requiring pharmacological management, ultimately leading to treatment discontinuation. To our knowledge, this is the first case report to demonstrate a successful mutation-driven therapeutic decision in this specific clinical setting of endometrioid adenocarcinoma, where the identification of a pathogenic *NF1* loss-of-function mutation provided the only viable targeted pathway in a tumor lacking standard biomarkers such as estrogen or progesterone receptor expression or microsatellite instability. This case serves as a paradigm for mechanism-driven precision oncology, illustrating that everolimus monotherapy can achieve durable clinical benefit in *NF1-*deficient recurrent endometrial cancer, with remission persisting even after treatment discontinuation. These findings highlight the clinical relevance of comprehensive molecular diagnostics and support the integration of personalized treatment strategies into modern gynecologic oncology.

## Introduction

Globally, the International Agency for Research on Cancer (IARC) under the World Health Organization (WHO) reported approximately 20 million new cancer cases and around 9.7 million cancer-related deaths in 2022. Corpus uteri neoplasms accounted for 420,242 new cases, representing approximately 2% of all cancer diagnoses that year with 97,704 deaths, corresponding to roughly 1% of all cancer-related deaths (1). Poland is among the countries with the highest cancer-related mortality. According to estimates from Polish National Cancer Registry (PNCR), in 2023 there were 6,161 new cases of corpus uteri cancer diagnosed in women, representing roughly 7% of all reported cancer incidents. In the same year, 2,109 deaths were attributed to this cancer, representing about 5% of all cancer-related deaths (2).

Endometrial cancer is a malignant neoplasm of the female reproductive system that develops in the mucosa lining the cavity of the uterus. The highest incidence is reported in perimenopausal and postmenopausal women, with a peak between the ages of 55 and 65. It is the most frequently diagnosed malignancy of the female reproductive tract in highly developed countries, a trend attributed to population aging and the increasing tendency of women to delay childbearing (3, 4). Histopathologically, endometrial cancer is classified into two main subtypes: type I (endometrial adenocarcinoma), which comprises approximately 80% of cases and type II (serous adenocarcinoma), although accounting for only about 15% of all reported cases is characterized by a significantly more aggressive clinical course. Type I tumors are typically associated with prolonged estrogen exposure. In contrast, the Type II tumors are not associated with unopposed estrogen stimulation and do not develop on the basis of endometrial hyperplasia (5).

Numerous factors contribute to the increased risk of endometrial cancer, including hypertension, type 2 diabetes, insulin resistance, and polycystic ovary syndrome (PCOS) as well as hormone replacement therapy (HRT). Obesity represents one of the most significant modifiable risk factors for endometrial cancer. The risk of developing endometrial carcinoma rises in a dose-dependent manner with increasing body mass index (BMI). Hormonal and reproductive factors such as early menarche, late menopause and nulliparity also increase the risk, whereas oral contraceptive use and early menopause have been shown to exert a protective effect (6, 7). It is also important to consider genetic predispositions, particularly mutations in genes such as *PTEN* which are found, among others, in Cowden syndrome. Furthermore, alterations in *PIK3CA* and *CTNNB1* have been associated with both the pathogenesis and elevated risk of endometrial cancer. Additionally, Lynch syndrome is the most common hereditary cause of endometrial cancer, in which pathogenic variants in one of the four mismatch repair genes (*MLH1, MSH2, MSH6*, or *PMS2*) increase the lifetime risk of endometrial cancer (4, 7). These molecular alterations disrupt normal cellular growth control, leading to uncontrolled proliferation and malignant transformation of the endometrial lining. Moreover, it is believed that women with a first-degree relative affected by endometrial cancer have a twofold increased risk of developing the disease, even in the absence of an identified pathogenic genetic variant (7).

## Case description

In March 2019, a 64-year-old woman with class I obesity, and a notable history of long-term smoking amounting to approximately 20 pack-years was admitted to the Department of Gynecology with suspected uterine malignancy. The patient underwent endometrial biopsy and was subsequently diagnosed with grade 1 (G1) endometrioid adenocarcinoma of the endometrium, stage IA according to the International Federation of Gynecology and Obstetrics (FIGO) 2009 classification. Preoperative pelvic MRI showed no suspicious lymphadenopathy. She underwent a total laparoscopic hysterectomy with bilateral salpingo-oophorectomy (TLH/BSO). Histopathological examination of the resected tissue confirmed the diagnosis of grade 1 (G1) endometrioid adenocarcinoma of the endometrium with myometrial invasion of less than 50% (FIGO 2009 IA). Importantly, both lymphovascular space invasion (LVSI) and perineural invasion (PNI) were identified. Immunohistochemical analysis revealed a lack of estrogen receptor (ER) and progesterone receptor (PR) expression but demonstrated p53 overexpression (p53+). Furthermore, the tumor was characterized by microsatellite stability (MSS) and absence of *POLE* mutations. The patient was then referred to the clinical oncologist for follow-up, which included physical examination every 3 months combined with CA125 screening and MRI of the abdomen and pelvis every 3 months.

In February 2022, a scheduled contrast-enhanced MRI examination detected a recurrent lesion at the vaginal apex measuring 11x8x17 mm [anteroposterior (AP) x transverse (TR) x craniocaudal (CC)] along with multiple peritoneal metastases. The largest lesion in the peritoneum measured 16x13 mm. Additionally, a solid lesion was identified in the rectus abdominis muscle, (47x33x40 mm). A concurrent chest CT scan showed no signs of metastatic disease. Laparoscopic cytoreductive surgery was performed and peritoneal metastases were resected.

In May 2022, a full-body contrast-enhanced computed tomography (CT) was performed which revealed multiple pulmonary metastases, as well as new soft-tissue lesions in the greater omentum, located in the mid-abdomen, among these the largest measured 32x28 mm (APxTR).

Due to the advanced dissemination of the neoplastic process, chemotherapy was initiated in May 2022 according to the TC regimen: carboplatin [area under the curve (AUC) 6 intravenous (IV)] and paclitaxel (175 mg/m2 IV) on day 1 every 21 days. At the beginning of the therapy the patient was in overall good condition, with an Eastern Cooperative Oncology Group (ECOG) performance status of 0. The overall tolerance of the TC chemotherapy was satisfactory, as no serious adverse events were observed. CT was performed after 3 cycles in which stable disease (SD) according to the response evaluation criteria in solid tumors (RECIST) 1.1 was described. Unfortunately, CT after completion of 6 cycles revealed progressive disease (PD), according to the RECIST 1.1, as the target lesions dimensions increased by 33%. Hence, in August 2022 the second-line chemotherapy with doxorubicin (60 mg/m2 IV) every 21 days and was initiated. The patient received six cycles of chemotherapy, complicated by the hematological toxicity after the third cycle, which included grade 2 anemia and grade 3 thrombocytopenia according to the CTCAE (Common Terminology Criteria for Adverse Events) v 5.0. Fortunately, CT after the completion of 6th cycle of doxorubicin showed complete response (CR) according to the RECIST 1.1. The patient was qualified for further oncological observation.

In June 2023, a follow-up MRI of the abdomen and pelvis revealed two hepatic lesions in segments VII and VIII, measuring 9.5x5 mm and 13x9 mm and a focal lesion measuring 11x10 mm (APxTR), implanted in the peritoneum. Due to the limited efficacy of third-line treatment options, enhanced molecular testing was performed using next-generation sequencing (NGS). A pathogenic somatic loss-of-function mutation in the *NF1* (neurofibromin 1) gene (*c.7759G>C*) was identified.

Consequently, the patient was qualified for targeted treatment with the mTOR kinase inhibitor, everolimus at the dose of 10 mg *per os* daily. In September 2023, after three months, follow-up MRI demonstrated a 50% reduction of hepatic lesions in VII and VIII segments which corresponds to the partial response (PR) according to RECIST 1.1 and complete resolution of peritoneal metastases. In June 2024, CR according to the RECIST 1.1. was documented (**Figure 1**).

**Figure 1:**
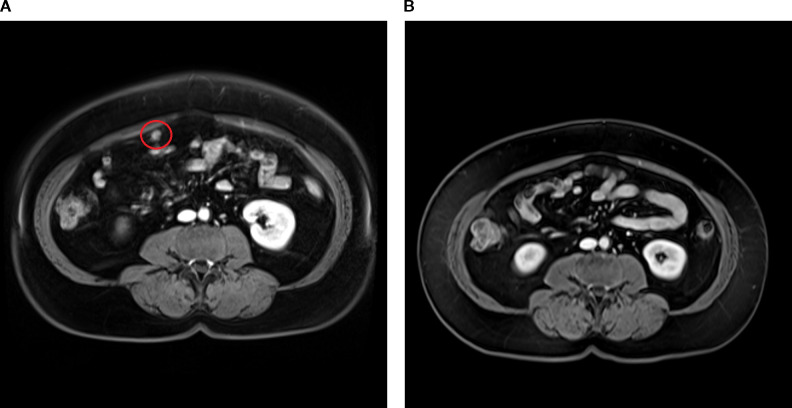
**(A)** abdomen MRI performed in June 2023. Abdomen MRI performed in June 2023 showing a focal lesion implanted in the peritoneum measuring approximately 11x10 mm (APxTR). **(B)** abdomen MRI performed in September 2023. Abdomen MRI performed in September 2023 shows complete regression of the metastatic lesion implanted in the peritoneum.

During the course of therapy, in July 2024 the patient developed significant side effects related to lipid metabolism. Laboratory tests revealed markedly elevated levels of total cholesterol, low-density lipoprotein (LDL) fraction and triglycerides, corresponding to grade 4 hyperlipidemia according to CTCAE v5.0 (**Table 1**). Atorvastatin at the dose of 20 mg per os daily was introduced and molecular therapy with everolimus was discontinued, resulting in normalization of lipid parameters after three months (**Table 1**). Fortunately, the termination of everolimus treatment did not cause progression of the disease. Complete remission has been sustained to date, with the most recent follow-up MRI performed in February 2026.

**Table 1:** Biochemical laboratory markers during and after discontinuation of everolimus therapy.

Biochemical laboratory marker	Prior treatment (June 2024)	During everolimus therapy (July 2024)	3 Months after the discontinuation of everolimus (October 2024)
Total cholesterol	182.0 mg/dL	1030.0 mg/dL	213.0 mg/dL
LDL-cholesterol	127.0 mg/dL	>600 mg/dL	129.0 mg/dL
HDL-cholesterol	54.0 mg/dL	99.0 mg/dL	51.0 mg/dL
Triglycerides	166.0 mg/dL	6679.0 mg/dL	164.0 mg/dL
Non-HDL cholesterol	128.0 mg/dL	931.0 mg/dL	162 mg/dL

## Discussion

Stage IA G1 endometrioid endometrial cancer is generally considered a low-risk group for recurrence, with reported rates of progression after radical surgery ranging from 5% to 10% (7, 8). According to the study by Nwachukwu et al., the risk factors for recurrence in patients with IA G1 tumors include: tumor size (≥2 cm), biopsy-to-surgery interval (≥6 months), surgical extent (hysterectomy vs hysterectomy/BSO), and myometrial invasion (9). In the presented case, the patient experienced a recurrence just over three years after undergoing TLH/BSO. Thus, it remains important to conduct regular oncologic observation and regular follow-up examinations of patients after completing oncologic treatment.

The *NF1* gene, located at 17q11.2, encodes neurofibromin, a tumor suppressor protein that functions as a GTPase-activating protein for rat sarcoma virus (RAS) protein. Neurofibromin accelerates the conversion of RAS bound to guanosine triphosphate (RAS-GTP) into its inactive form bound to guanosine diphosphate (RAS-GDP), thereby providing negative regulation of the RAS signaling pathway. Loss-of-function mutations in *NF1* result in constitutive RAS activation and sustained stimulation of downstream effectors, including the RAF (rapidly accelerated fibrosarcoma)-MEK (mitogen-activated protein kinase kinase)-ERK (extracellular signal-regulated kinase) cascade and the PI3K (phosphatidylinositol 3-kinase)-AKT (protein kinase B)-mTOR (mechanistic target of rapamycin) pathway. This, in turn, can lead to enhanced cellular proliferation, increased survival, and decreased sensitivity to apoptotic cues, thereby disrupting normal cell cycle control and homeostatic mechanisms. Collectively, these effects create a cellular environment conducive to malignant transformation, promoting tumor initiation and progression by supporting uncontrolled growth and evasion of cell death mechanisms (10, 11). Everolimus, an mTOR inhibitor, directly targets this aberrant signaling, providing a rationale for its use in tumors harboring somatic *NF1* loss-of-function mutations. Everolimus inhibits the RAS-RAF-MEK-ERK-AKT-mTOR pathway as illustrated in **Figure 2**.

**Figure 2:**
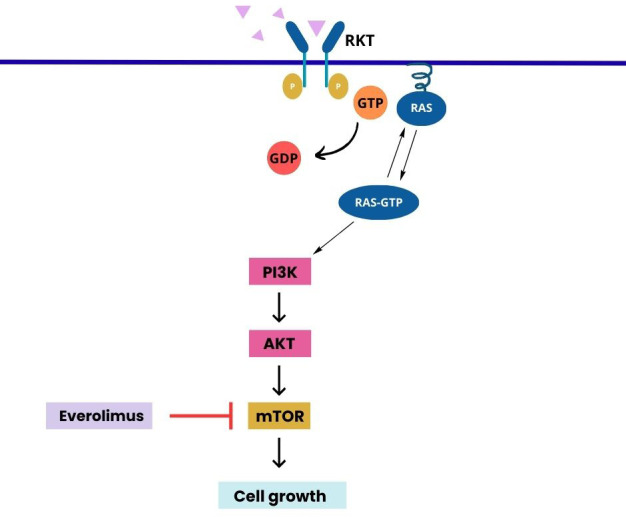
Everolimus mechanism of action. Everolimus mechanism of action in the RAS-RAF-MEK-ERK-AKT-mTOR signaling pathway.

In a study by Lim et al., NGS of tumor tissue from patients who achieved unusually prolonged clinical benefit from everolimus revealed a significant enrichment of mutations activating the mTOR pathway, including somatic *NF1* loss-of-function mutations. These findings indicate that *NF1*-deficient tumors, regardless of their tissue of origin, may exhibit increased biological susceptibility to mTOR inhibition. Therefore, the presence of a somatic *NF1* loss-of-function mutation provides a strong mechanistic rationale for considering everolimus as a targeted therapeutic option, particularly in settings where conventional systemic therapies show limited activity (12).

In the presented case, a pathogenic loss-of-function mutation was identified in the *NF1* gene. Importantly, *NF1* mutations are not commonly associated with endometrial cancer. To date, only a limited number of cases have been reported linking *NF1* mutations with the development of endometrial carcinoma, highlighting the rarity and atypical nature of this presentation (13). In 2018 O’Mara et al., identified nine genetic loci, including *NF1*, potentially linked to endometrial cancer susceptibility - none of which had previously been recognized as significant risk factors (14).

According to the current National Comprehensive Cancer Network (NCCN) guidelines, systemic hormonal therapy for recurrent or metastatic endometrial carcinoma is primarily recommended for lower-grade endometrioid histologies, particularly in patients with low tumor burden or a slowly progressive, indolent disease course. Among available therapeutic options, the combination of everolimus and letrozole is recommended, regardless of the *NF1* mutational status. The strategy utilizes mTOR pathway inhibition (everolimus) together with estrogen synthesis suppression via aromatase inhibition (letrozole), providing a biologically synergetic effect that may enhance tumor growth control in hormonally responsive disease (15). In the phase II, single-arm clinical trial by Slomovitz et al., the efficacy of everolimus and letrozole was evaluated in patients with progressive or recurrent, previously treated endometrioid endometrial carcinoma. The study enrolled patients with progressive or recurrent endometrial carcinoma who had undergone up to two prior lines of chemotherapy. Histologic confirmation of the diagnosis was mandatory, while patients with carcinosarcoma or sarcoma were excluded. Additional eligibility criteria included ECOG performance status 0–2 and no history of any other invasive malignancies within the past five years. In this study, CR was observed in 9 patients (25.7%), while PR was reported in 2 patients (5.7%). Moreover, the median overall survival (OS) was 14 months, and the median progression-free survival (PFS) was 3.0 months (16). However, in the presented case, the tumor was characterized by a lack of ER and PR receptor expression. This negative receptor status suggests that the addition of letrozole might have offered limited clinical benefit, as the disease appeared to lack a primary hormonal driver. Furthermore, the identification of MSS was a crucial factor. While immune checkpoint inhibitors have revolutionized the treatment of mismatch repair-deficient (dMMR) endometrial cancers, their efficacy in MSS tumors is significantly lower, although some patients may still achieve clinical benefit (7). In this case, immunotherapy was considered but not pursued, as it was not reimbursed in Poland for this indication at the time. Unfortunately, the patient could not cover the substantial costs of private treatment. The exclusion of both hormonal and immunotherapy options further narrowed the therapeutic field, leading to the selection of targeted therapy based on the identified *NF1* loss-of-function mutation.

Everolimus monotherapy in the treatment of endometrial cancer, as in our patient, was studied in a phase II, single-arm trial by Ray-Coquard et al. The study was conducted among 44 patients with advanced or metastatic endometrial cancer (endometrioid, serous and other) resistant to one or two prior lines of chemotherapy, with at least one being platinum-based. One patient was excluded from the analysis due to an unknown death cause. PR was observed in 9% of patients, while stable disease was achieved in 27% of cases. In the overall study population, the median overall survival (OS) was 8.1 months, and the median progression-free survival (PFS) was 2.8 months. Among the 43 patients assessed for safety, all experienced adverse events, most of which were grade 1-2. The most frequently observed grade 3–4 adverse events included fatigue (42%), anorexia, and infections. Metabolic abnormalities were common, as hypercholesterolemia (81%), hypertriglyceridemia and hyperglycemia occurred in 81%, 69% and 61% respectively. Mucositis was the most frequent reason for the dose reductions, being responsible for 39% of reductions and 23% of dose interruptions (17).

In our patient, given the mechanism of action of everolimus, in the light of the presence of *NF1* loss-of-function mutation and taking into account the results of the phase II study by Ray-Coquard et al., we decided to introduce everolimus in the treatment of our patient. This unorthodox approach proved to be successful as demonstrated by the complete regression of target lesions in response to everolimus therapy, which represents a particularly remarkable and clinically significant outcome. Moreover, a sustained response lasting more than 1.4 years after everolimus discontinuation is a rare phenomenon, given that in metastatic malignancies withdrawal of targeted therapies usually results in rapid disease progression. This “disease flare” phenomena have been observed with other targeted agents, including EGFR and ALK tyrosine kinase inhibitors, where abrupt cessation of the treatment can trigger rapid tumor growth (18).

Everolimus, like any pharmacological agent, carries the risk of adverse effects In the phase III randomized trial of everolimus versus placebo in patients with metastatic renal cell carcinoma, the most frequent adverse events of any grade included stomatitis (44%), fatigue (37%), rash (31%), diarrhea (30%), anorexia (27%), and hypercholesterolemia (26%), while the most common grade 3–4 events were anemia (7%), infections (7%), dyspnea (6%), hyperglycemia (5%), stomatitis (4%), and pneumonitis (3%). Permanent treatment discontinuation due to adverse events occurred in 14% of everolimus-treated patients versus 3% in the placebo arm, indicating that although toxicities were common, most were low-grade and manageable (19). According to a meta-analysis by Arena et al., which focused on side-effects of everolimus therapy, hypercholesterolemia was reported in 1078 out of 5349 patients from included studies, corresponding to an incidence rate of 20.2%, with grade 3 and 4 severity observed in only 1.3% of cases (20). In our patient’s case, critically elevated levels of total cholesterol (1030 mg/dL) and triglycerides (6679 mg/dL) were documented, values corresponding to grade 4 toxicity, indicating severe, life-threatening consequences requiring urgent clinical intervention according to CTCAE v5.0. Remarkably, 3 months after introducing atorvastatin a full normalization of the lipid profile was observed. This rapid biochemical recovery is particularly striking, as prior meta-analyses have reported lipid normalization typically occurring no earlier than six months after treatment discontinuation (20).

## Conclusion

This case report underscores the importance of individualized care in oncology, encompassing all stages of the clinical pathway - from diagnosis, through selection of treatment modalities, to extended post-therapeutic follow-up. To our knowledge, this is the first case report to demonstrate a successful mutation-driven therapeutic decision in this specific clinical setting of endometrioid adenocarcinoma, where the identification of a pathogenic *NF1* loss-of-function mutation (*c.7759G>C*) via NGS provided the only viable targeted pathway in a tumor lacking standard biomarkers such as ER/PR expression or microsatellite instability. Modern cancer treatment increasingly relies on a personalized, patient-centered approach, in which therapeutic strategies are tailored to the individual clinical and molecular profile of each case. This case exemplifies this paradigm, being the model example of mechanism-driven decision making. As illustrated by this case, everolimus monotherapy may achieve meaningful clinical benefit in tumors with *NF1* loss-of-function mutation. However, it should be emphasized that further prospective studies are needed to identify the molecular determinants of response to everolimus in endometrial cancer.

## Data Availability

The original contributions presented in the study are included in the article/supplementary material. Further inquiries can be directed to the corresponding author.
